# Supplementary material for: Change in Growth Status and Obesity Rates among Saudi Children and Adolescents Is Partially Attributed to Discrepancies in Definitions Used: A Review of Anthropometric Measurements
Source: Healthcare (Basel). 2023 Apr 1;11(7):1010. doi: 10.3390/healthcare11071010 (PMC10094271; doi:10.3390/healthcare11071010)
Supplement: Supplementary file 1 [file healthcare-11-01010-s001.zip › healthcare-2240990-Supplementary.pdf]

healthcare

Supplementary Material

Change in growth status and obesity rates among Saudi children and adolescents is partially attributed to discrepancies in definitions used: A review of anthropometric measurements.

Essra Noorwali *, Abeer Aljaadi, Hala Al-Otaibi

*** Correspondence:** eanoorwali@uqu.edu.sa

Table S1. Systematic search terms using MeSH for the literature review

1.	Body weight
2.	Body weight.tw.
3.	(Body adj2 weight*).tw.
4.	exp body weight
5.	Body composition
**6.**	**or/1-5 [body weight]**
7.	Body height
8.	Body height.tw.
9.	(Body adj2 height*).tw.
10.	exp body height
**11.**	**or/7-10 [body height]**
12.	Waist-height ratio
13.	Waist-height ratio.tw.
14.	(Waist adj2 height ratio*).tw.
15.	exp waist-height ratio
**16.**	**or/12-15 [waist-height ratio]**
17.	Waist circumference
18.	Waist circumference.tw.
19.	(Waist adj2 circumference*).tw.
20.	exp waist circumference
21.	exp waist-hip ratio
**21.**	**or/17-21 [waist circumference]**

22.	exp anthropometry
23.	exp body weights and measures
24.	exp body size
25.	exp head circumference
**26**	**or/22-25 [anthropometry]**
27.	Growth charts
28.	Growth charts.tw.
29.	(Growth adj2 charts*).tw.
30	exp growth charts
31	Reference growth curves
32	Reference standards
33	Diagnostic reference levels
**34.**	**or/27-33 [growth charts]**
**35.**	**exp newborn/or exp child/or exp adolescent**
36.	Saudi Arabia
37.	Saudi Arabi. tw.
38.	(Saudi adj2 Arabia*).tw.
39.	exp Saudi Arabia
**40.**	**or/36-39 [Saudi population]**
**41.**	**6 and 11 and 16 and 21 and 26 and 34 and 35 and 40**

Tw, text word search in title or abstract fields.
Adj2, terms within two words of each other (any order)
Explosion of a term means, that by adding **"exp"** to your keyword ("exp keyword") all the lower "branches" of this specific term are automatically included in the search
* Truncation (symbols may differ depending on the database)

Table S2. Saudi children/ adolescent studies measuring anthropometrics before 1990.

No.	Author, Year (reference)	Regions	Population age	Sample n	Anthropometrics assessed	Anthropometric assessment definition (e.g., WHO, CDC, NCHS..)	Comments
					**Children studies**		
**1.**	Wirths et al. 1977 [1]	Riyadh, Jeddah,	6-9 years	341	Weight, height, skinfold-thickness, circumferences and		Article in German- translated by author EN

		Abha and Dammam			other body-size measurements		
**2.**	Sebai et al. 1981[2]	Wadi Turaba	<5 years	314	Weight, height, head and chest circumference	Harvard standard 50th percentile	Anthropometric measurement method mentioned
**3.**	Sebai et al. 1981 [3]	Khulais villages	Primary school children	280	Weight, height, and head and arm circumferences	Not mentioned	Anthropometric measurement method not mentioned
**4.**	Abdullah et al. 1982[4]	Central-2 rural villages	0-5 years	337	weight-for-age, weight-for-height and height-for-age	Based on several references	Anthropometric method mentioned briefly
**5.**	Taha et al 1984 [5]	Riyadh	infants	967	Weight, height, head circumference	Compared to British infants	Anthropometric measurement method mentioned.
**6.**	**Al-Frayh et al 1987** [6]	**Riyadh**	**1 month-5 years**	**6400**	**Weight, height, head, chest circumference, triceps skin fold and mid arm circumference**		**The objective of this study was to construct physical growth standards for Saudi infants and preschool children by identifying and measuring a statistical sample of healthy and well -fed infants and children. This is the first published growth data from Riyadh population. Anthropometric measurement method not mentioned. Information on feeding pattern was collected however, the questions were not clear. In addition, there was no definition of "healthy" and "well-fed" infants and children.**
**7.**	Serenius 1988 [7]	Riyadh	0-36 months	315	Weight, height, weight for height and head circumference, skinfold thickness	NCHS and other references	Anthropometric measurement method mentioned
**8.**	Serenius and Sawilem 1988[8]	Riyadh	1-71 months		Weight, height and weight for height, triceps skinfold thickness	NCHS /WHO and other references	Anthropometric measurement method mentioned
**9.**	Serenius et al 1988 [9]	3 regions	0-71 months	842	Weight, height, weight for height and head circumference, upper arm muscle and fat areas	NCHS/WHO and other references	Anthropometric measurement method mentioned
**10.**	Al-Othaimeen and	Eleven health centers	0-3 years old	767	Height for age, weight for height	NCHS	Regions of SA not mentioned. 538 participants were assessed for nutritional status. Total

	Villanueva 1988 [10]						subsample suffered mild stunting. Anthropometric measurement method not mentioned
**11.**	Al-Othaimeen et al 1988 [11]	Four regions	0-6 years	849	Height for age, Weight for height	NCHS	No access to the paper just the abstract.<br>Mild to moderate stunting for most age-sex groups.
**12.**	Krueger N, 1988 [12]	Najran	Newborn infants	1436	Weight, crown-to-heel<br>length, and head circumference	Not mentioned	Anthropometric measurement method briefly mentioned with no reference
**13.**	Baner and Al-Frayh 1989[13]	Not mentioned	<6 years	4614	Studied the relationship between average height and weight	Ehrenberg's approach	This is a letter to the editor. Anthropometric measurement method based on [6].
**14.**	Rasheed et al 1989 [14]	urban area of Thugba, Al-Khobar	Primary school girls	285	Weight, height	NCHS	Anthropometric measurement method mentioned
**15.**	Wong and Al-Frayh 1989 [15]	Riyadh	Infants born within 24 hours	4497	Weight, height, head, chest and mid arm circumference	Not mentioned	Anthropometric measurement method mentioned. Effects of consanguineous marriage on anthropometrics was studied

BOLD highlighted rows: studies contributed to the establishment/adjustment of Saudi growth charts for children/adolescents or specific cut-offs or studied the trend of growth in representative samples or adjusted the international curves to be used in Saudis.

Legend: BMI: Body mass index (may refer to z-scores of BMI), CDC: Centers for Disease Control, FM: Fat mass, IOTF: International Obesity Task force, LBW: low birth weight, LMS: Lambda-Mu-Sigma, NCHS: National Center for Health Statistics, NHANESI: National Health and Nutrition Examinations Survey I, NICHD: National Institute of Child Health and Human Development, SA: Saudi Arabia, SAD, Sagittal abdominal diameter, WHO: World Health Organization

Table S3. Guidelines and recommendations for updating the Saudi Growth charts and to assess the quality of anthropometric survey data based on the WHO recommendations[16].

**Children from 0-5 years old**
1. The sample should be nationally representative and allows for regional and district level estimates. This will show the differences between regions that may be due to several factors.
2. From the previous point, since several factors impact anthropometric measurements, disaggregation categories such as (wealth, maternal education, dietary intake, physical activity…etc.) may be used to assist in determining the causes of anthropometric changes and thus in intervention methods.
3. Selecting the primary sampling units (PSU) with probability proportional to size (PPS) by an individual working in the national statistical office. All households in Saudi Arabia should be included regardless of the nationality (Saudis, and non-Saudis) because the aim is the report to the progress toward the Sustainable Development Goals (SGDs) and therefore no one should be left out. In addition, because lifestyle and habits of non-Saudis living in Saudi Arabia may be similar to Saudis[17]. However, the studies conducted previously were limited to Saudis only making it difficult to compare between anthropometrics of Saudis and non-Saudis living in Saudi Arabia.
4. Tracking trends of stunting and overweight using the Global Targets Tracking Tool available on the WHO website may help Saudi Arabia set national targets and monitor progress towards World Health Assembly targets three of which are stunting, overweight and wasting.
5. Several points need to be checked to assess the quality of anthropometric survey data to generate accurate child malnutrition status. These points are summarized in section 3.1 in the WHO guideline [16] Several points may not have been applied when the Saudi Growth charts survey were created.<br><br>a. **<u>Sex ratio</u>** which is the proportion of males to females for specific age groups in a given population. The sex ratio of the survey should be compared to a reference

(expected sex ratio) to identify selection bias. The WHO guideline provides the United Nations Population Division World Population Prospects as a potential reference which estimates sex ratios using smoothed distributions on expected sex ratios at birth and mortality levels by country and year for different age groups. In addition, other national sources such as the latest censuses or other nationally representative survey reports for the same time period as the survey can be used. Recent literature has shown that the sex ratio has changed in Saudi Arabia [18][19].

b. **Age heaping** which is the unexpected distribution of observations for specific ages and/or months of birth need to be considered. The reason is that unequal distributions between single-year age groups or too few children in a specific age group may be related to selection bias. How to calculate, present and interpret the data is reported in the WHO guideline[16].

c. **Digit preference** which refers to an unexpected distribution of digits in weight and length/height measurements. This is a significant facto r to check because a digit preference for terminal digits of weight will result in greater inaccuracies when estimating prevalence for weight-for-age z-score (WAZ) and weight-for height z-score (WHZ) than a digit preference for terminal digits of length/height would have on height-for age z-score (HAZ) or weight for height z-score (WHZ). Consequently, prevalence of wasting, stunting, overweight and obesity may be misreported.

d. **Z- score values** that fall outside a specified range need to be checked because they may be due to poor measurement, inaccurate date of birth or data recording errors.

e. **Standard deviations (SD) of z scores** quantifies the amount of variability in a dataset. The WHO recommend using SD as a data quality criterion because prevalence estimates of stunting, wasting and overweight are dichotomous variables which measure the percentage of children with z-score values beyond a specified cut-off (e.g., <-2 SD for wasting or stunting, >+2 SD for overweight). If the SD is artificially inflated as a result of poor-quality data, prevalence estimates are therefore likely to be overestimated. However, the cut-offs provided by the WHO need to be revised for various reasons including: the surveys used were not all nationally representative therefore, they should reflect nationally representative surveys among populations with varying degrees of malnutrition. The second reason that the SD cut-offs provided by the WHO need to be revised is that they were based on the distribution of z-scores calculated

using the NCHS/WHO reference for child growth which was replaced in 2006 with the WHO Child Growth Standards in use today.<br><br>f. **Shape of the frequency distribution** of WAZ, HAZ and WHZ is a significant factor to be assessed because it provides insights into the data quality and survey population. Prevalence of severe forms of malnutrition (e.g., severe stunting, overweight and obesity) may cause probability distribution that can deviate from normal distribution. Further research needs to be carried out before practical guidance on interpreting the shape of the distribution curve in any given survey can be provided. Nevertheless, comparisons of distributions between groups can provide hints that can help interpretation.
**Children aged >5 years old**
For children >5 years of age, the same cleaning approach used for the construction of the WHO Child Growth Standards for children < 5 years old (cross-sectional component) was applied to avoid the influence of unhealthy weights-for-height. Therefore, the same points mentioned earlier for children <5 years old need to be considered to prevent false impressions of good percentage of the population being in a healthy condition due to an increase in the normal weight curves.<br><br>However, it is important to note that the sample used to construct the growth charts for children aged >5 years were collected solely from the American population and no Arabic countries were included making it necessary for Saudi Arabia to update their growth charts for this age group. Several countries had created their growth charts and compared them with the WHO growth charts including Korea[20], Spain[21] and Iran[22]. Some of the methods used when creating their growth charts may be applied to when updating the Saudi Growth charts.

References:

1. Wirths, W.; Hamdan, M.; Hayati, M.; Rajhi, H. [Nutritional status, food consumption and food supply of studies in Saudi Aribia. I. Anthropometric data]. *Z. Ernahrungswiss.* **1977**, *16*.

2. Sebai, Z.A.; Reinke, W.A. Anthropometric measurements among pre-school children in Wadi Turaba, Saudi Arabia. *J. Trop. Pediatr.* **1981**, *27*, doi:10.1093/tropej/27.3.150.

3. SEBAI, Z.A.; SABAA, H.M.A.; SHALABI, S.; BAYOUMI, R.A.; MILLER, D. Health in

Khulais villages, Saudi Arabia: An educational project. *Med. Educ.* **1981**, *15*, doi:10.1111/j.1365-2923.1981.tb02494.x.

4. Abdullah, M.A.; Swailem, A.; Taha, S.A. Nutritional status of preschool children in central Saudi Arabia. *Ecol. Food Nutr.* **1982**, *12*, doi:10.1080/03670244.1982.9990702.
5. TAHA, S.A.; ABDULLAH, M.A.; JOWDA, M.S.; AKBAR, J.U. Size at birth of live-born Saudi infants. *BJOG An Int. J. Obstet. Gynaecol.* **1984**, *91*, doi:10.1111/j.1471-0528.1984.tb04737.x.
6. Al-Frayh, A.R.; Jabar, F.A.; Wong, S.S.; Wong, H.Y.H.; Bener, A. Growth And Development Of Saudi Infant And Pre-School Children. *J. R. Soc. Promot. Health* **1987**, *107*, doi:10.1177/146642408710700107.
7. SERENIUS, F. Attained Grawth of Privileged Saudi Children During the First Three Years of Life. *Acta Paediatr.* **1988**, *77*, 80–92, doi:10.1111/j.1651-2227.1988.tb10873.x.
8. Serenius, F.; Swailem, A.R. Growth and nutritional status of less privileged urban children in Saudi Arabia. *Acta Paediatr. Scand. Suppl.* **1988**, *77*, doi:10.1111/j.1651-2227.1988.tb10874.x.
9. Serenius, F.; Fougerouse, D.; Sebai, Z. Growth and nutritional status of rural preschool children in Saudi Arabia. *Acta Paediatr. Scand. Suppl.* **1988**, *77*, doi:10.1111/j.1651-2227.1988.tb10875.x.
10. Al-Othaimeen, A.I.; Villanueva, B.P. The effects of prolonged breast-feeding on the nutritional status of Saudi Arabian children. *Ann. Saudi Med.* **1988**, *8*, doi:10.5144/0256-4947.1988.97.
11. AL-OTHAIMEEN, A.; SAWAYA, W.; TANNOUS, R.; VILLANUEVA, B. A nutrition survey of infants and preschool children in Saudi Arabia. *Saudi Med. J.* **1988**, *9*.
12. Krueger, N.W. Size of birth in Najran, Saudi Arabia. *Ann. Saudi Med.* **1988**, *8*, doi:10.5144/0256-4947.1988.113.
13. Bener, A.; Al-Frayh, A. Relationships Between Height and Weight of Saudi Children. *Ann. Saudi Med.* **1989**, *9*, 92–93, doi:10.5144/0256-4947.1989.92.
14. Rasheed, P.; Al-Yousef, N.; Al-Dabal, B. Nutritional profile of Saudi primary schoolgirls in an urban region. *Ann. Saudi Med.* **1989**, *9*, doi:10.5144/0256-4947.1989.371.
15. SAEDI-WONG, S.; AL-FRAYH, A.R. Effects of Consanguineous Matings on

Anthropometric Measurements of Saudi Newborn Infants. *Fam. Pract.* **1989**, *6*, 217–220, doi:10.1093/fampra/6.3.217.

16. World Health Organization and the United Nations Children's Fund (UNICEF) *Recommendations for data collection, analysis and reporting on anthropometric indicators in children under 5 years old. Geneva: World Health Organization and the United Nations Children's Fund (UNICEF)*; 2019;
17. Alzeidan, R.A.; Rabiee, F.; Mandil, A.A.; Hersi, A.S.; Ullah, A.A. Changes in dietary habits, physical activity and status of metabolic syndrome among expatriates in Saudi Arabia. *East. Mediterr. Heal. J.* **2017**, *23*, doi:10.26719/2017.23.12.836.
18. Khraif, R.; Salam, A.A.; Potty, R.S.; Aldosari, A.; Elsegaey, I.; AlMutairi, A. Variations in basic demographics consequential to population size of governorate in Saudi Arabia. *Springerplus* **2016**, *5*, doi:10.1186/s40064-016-3126-0.
19. Khraif, R.M.; Salam, A.A.; Nair, P.S.; Elsegaey, I. Migration in Saudi Arabia: Present and prospects. In *India's Low-Skilled Migration to the Middle East: Policies, Politics and Challenges*; 2019.
20. Kim, J.H.; Yun, S.; Hwang, S.S.; Shim, J.O.; Chae, H.W.; Lee, Y.J.; Lee, J.H.; Kim, S.C.; Lim, D.; Yang, S.W.; et al. The 2017 Korean national growth charts for children and adolescents: Development, improve ment, and prospects. *Korean J. Pediatr.* 2018, *61*.
21. Pérez-Bermejo, M.; Alcalá-Dávalos, L.; Pérez-Murillo, J.; Legidos-García, M.E.; Murillo-Llorente, M.T. Are the Growth Standards of the World Health Organization Valid for Spanish Children? The SONEV Study. *Front. Pediatr.* **2021**, *9*, doi:10.3389/fped.2021.700748.
22. Hosseinpanah, F.; Seyedhoseinpour, A.; Barzin, M.; Mahdavi, M.; Tasdighi, E.; Dehghan, P.; Momeni Moghaddam, A.; Azizi, F.; Valizadeh, M. Comparison analysis of childhood body mass index cut-offs in predicting adulthood carotid intima media thickness: Tehran lipid and glucose study. *BMC Pediatr.* **2021**, *21*, doi:10.1186/s12887-021-02963-y.
